# Role of Video-Assisted Thoracoscopic Surgery in Acute Empyema in Children: A Retrospective Analysis

**DOI:** 10.7759/cureus.99778

**Published:** 2025-12-21

**Authors:** Harish C Tudu, Vedaant Parekh, Subrat Mohanty, Shweta Poduval, Hema Varun Manne, Sruti Mohanty, Pradeep K Jena, Varsha Madhavnarayan Totadri

**Affiliations:** 1 Paediatric Surgery, Kalinga Institute of Medical Sciences, Bhubaneswar, IND; 2 General Surgery, Kalinga Institute of Medical Sciences, Bhubaneswar, IND

**Keywords:** minimally invasive surgery, paediatric empyema, pleural effusion, thoracoscopy, vats

## Abstract

Background

Acute empyema is a serious complication of paediatric pneumonia that often necessitates surgical intervention in advanced stages. Video-assisted thoracoscopic surgery (VATS) has emerged as one of the commonly used modalities of surgical management of empyema, due to its minimally invasive nature and favorable outcomes. The study aims to evaluate the efficacy and outcomes of VATS in the management of Stage II and III acute empyema in children at a single tertiary care center.

Methods

This retrospective study analyzed 22 paediatric patients with radiologically confirmed Stage II or III empyema who underwent VATS between January 2022 and March 2025. Preoperative characteristics, intraoperative findings, postoperative complications, and follow-up outcomes were assessed with appropriate statistical methods.

Results

Among 22 patients, the majority (n=15, 68.2%) had right-sided empyema. Common symptoms included fever in all the patients (100%) and cough in 19 (86.4%), with a mean symptom duration of 14.5 days. Preoperative intercostal drain (ICD) insertion was performed in 13 (59.1%) cases. VATS was successful in 19 (86.4%) patients, while three (13.6%) required conversion to thoracotomy. The mean operative time was 96.1 minutes, and the average blood loss was 60.2 mL. Postoperatively, 10 (45.5%) experienced fever, and two (9.1%) had air leaks. At the three-month follow-up, all patients showed complete radiological resolution with no residual or recurrent empyema.

Conclusion

VATS is a safe and effective surgical option for paediatric empyema unresponsive to medical therapy. Early intervention is associated with favorable recovery, minimal complications, and excellent long-term outcomes. VATS should be considered the primary surgical approach for Stage II and III empyema in children.

## Introduction

Pleural effusion secondary to bacterial pneumonia is a complication which is referred to as parapneumonic effusion. This effusion, if left untreated or not resolving with medication, progresses to a grossly purulent collection in the pleural space known as acute empyema. This progression represents a series of pathological changes that, if not properly treated, can lead to significant morbidity [1]. 

Empyema occurs in three recognised stages. The first exudative stage involves the buildup of sterile fluid in the pleural space. Without prompt treatment, this can move to the fibrinopurulent stage, where fibrin builds up, bacteria invade, and septation occurs, resulting in localised fluid collections within the pleural space. If left untreated or treated inadequately, it can progress to the organising or chronic phase. This phase is marked by the change of fibrin exudates into a thick fibrous layer between the visceral and parietal pleura, leading to severe pleural adhesions and difficulty in lung re-expansion [2]. 

The early stages of empyema in children are generally treated with an intercostal drain (ICD) to help with clearance. However, a few cases (Stage III empyema) do require surgery, especially when there is no improvement in clinical signs or imaging results. Recently, video-assisted thoracoscopic surgery (VATS) has become the preferred method over open thoracotomy, due to its minimally invasive nature, better recovery outcomes, and shorter hospital stays [3]. Intrapleural fibrinolytic therapy has also been used to treat empyema. This therapy aims to break up septations and improve drainage. However, the response to fibrinolytics can be unpredictable. Difficulties in evaluating resolution and the risk of leftover disease or recurrence may require further surgical treatment [4]. At our center, VATS is the preferred surgical method for treating acute, unresolved empyema in children. 

The aim of this study was to assess the role of VATS as a modality for the management of acute empyema in the paediatric population. We focused on identifying preoperative, intraoperative, and postoperative factors that might affect surgical results. Additionally, the study aimed to assess long-term postoperative outcomes, including the rate of leftover disease, recurrence of empyema, and the development of noticeable restrictive lung disease during routine follow-up.

## Materials and methods

Study design and study setting

This was a retrospective study conducted to assess the role of VATS as a modality for management of acute empyema in the paediatric population at the Department of Paediatric Surgery in Kalinga Institute of Medical Sciences and Pradyumna Bal Memorial Hospital, Bhubaneswar, Odisha, India, between January 2022 and March 2025.

Eligibility criteria

Inclusion Criteria

All children (age ≤ 18 years) who presented to the department during the study period with the diagnosis of stage II (fibrinopurulent stage) or stage III (organising stage) empyema, identified clinically or radiologically following pneumonia, and underwent VATS for the same, were included in the study. Additionally, inclusion required non-resolution of stage I empyema on chest X-ray 48 hours after insertion of an intercostal drainage tube.

Exclusion Criteria

Patients older than 18 years of age, those with incomplete documentation of operative or postoperative details, those who underwent VATS for indications other than acute empyema, cases lost to follow-up, and patients presenting with residual empyema after undergoing VATS or thoracotomy at an outside hospital were excluded from the study.

Sample size

**Figure 1:**
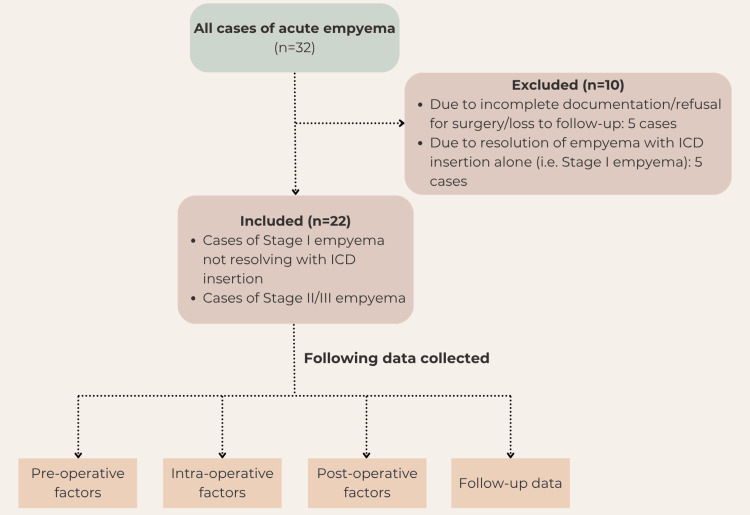
Flow chart illustrating the patient selection process ICD: intercostal drain

All eligible patients were included in the study. Based on the inclusion and exclusion criteria for the study and enrollment over three years, this included a sample size of 22 children. Figure 1 illustrates the patient selection process used in this study.

Study procedure

Data of patients presenting to the Department of Paediatric Surgery, Department of Paediatric Medicine, and Department of Paediatric Critical Care at Kalinga Institute of Medical Sciences and Pradyumna Bal Memorial Hospital, Bhubaneswar, with pneumonia and subsequent acute empyema, proven on radiological imaging, undergoing VATS, and satisfying the inclusion and exclusion criteria were analysed.

A variety of preoperative factors were analysed, including prior comorbidities, presenting symptoms and their duration, duration prior to hospitalisation, duration between hospitalisation and surgery, and whether or not ICD insertion and/or intubation were required before surgery. The current indications for VATS in our centre are as follows: (i) Non-resolution of symptoms (fever, respiratory distress, chest pain), despite ICD insertion, (ii) Presence of thickened or loculated collection in the pleural cavity documented on ultrasound or computed tomography (CT) of the thorax, (iii) Non-expansion of the affected lung with persistent collection idespite medical management and ICD in situ, (iv) Presence of collection within interlobar fissure, and (v) Stage II and III empyema.

ICD was inserted only in select cases preoperatively. The main indication was children who presented with acute empyema (usually, stage I radiologically) with significant respiratory distress, not improving with medical management. For patients in a stable state with organising or already organised empyema, which would not benefit from ICD insertion, preoperative ICD insertion was not done.

All VATS procedures undertaken were performed by paediatric surgeons with experience in thoracoscopic surgery. The surgical procedure and pre-and postoperative management also remained standardised across the department to ensure uniformity in management. After induction of general anaesthesia, the patient was positioned in a lateral decubitus position with the affected side up. Axillary rolls and other cushions were given as appropriate. In anticipation of significant blood loss, intraoperative blood transfusion was initiated at the time of incision. 

For patients who had preoperative ICDs inserted, the ICDs were removed, and a 10 mm camera port was inserted through the previous ICD site. For patients who did not require prior ICD insertion, the initial 10 mm camera port was placed in the fifth or sixth intercostal space in the mid-axillary line (at least one intercostal space below the inferior angle of the scapula). Following insertion of the first port, a camera was inserted, and intraoperative findings were noted. Carbon dioxide pneumothorax was created, and under vision, a working port of either 5 mm or 10 mm was inserted. The location of the second port was one that would maximise the ease of clearing the empyema, and at least two intercostal spaces below the first port. Due to the non-availability of a thoracoscope in our institute, we used the 5 mm and 10 mm laparoscopic ports for our procedures. However, this difference in instruments has not contributed to any intraoperative difficulty for our surgeons.

**Figure 2:**
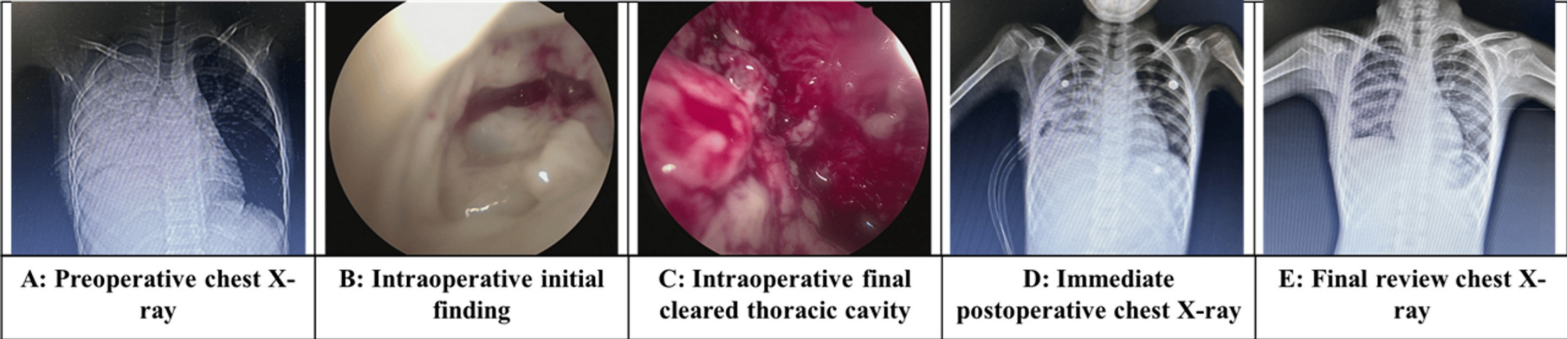
Composite image showing sequential stages of management Source: Medical Records Department of Kalinga Institute of Medical Sciences and Pradyumna Bal Memorial Hospital; Data of patients included in the study, who underwent video-assisted thoracoscopic surgery for acute empyema with parental consent

Intraoperative findings were noted as well as associated intraoperative complications, including but not limited to conversion to a thoracotomy. At the time of surgery, samples of the pus/pus flakes were collected and sent for microbiological and histopathological evaluation, the results of which were also analysed in our study. Frank pus, if present, was suctioned out. Fibrotic adhesions were cleared with instruments such as the suction rod and pus flakes, and these adhesions were cleared out of the pleural cavity using atraumatic cup forceps. Clots were suctioned out, and haemostasis was checked. Once satisfactory clearance of the pleural debris was achieved, the ports were removed under vision, and two ICDs were inserted within the pleural cavity. Based on the anatomical extent of the empyema, these ICDs would either be anterior/posterior or superior/inferior. ICDs were fixed to the skin with non-absorbable sutures, connected to an underwater seal bag, and a secure airtight dressing was applied over the ICD sites. Air column movement and the presence of any air leak were observed prior to shifting the patients to the ICU postoperatively. The chronological sequence of imaging and intraoperative findings is depicted in Figure 2, outlining the preoperative X-ray, intraoperative observations, and postoperative radiographic outcomes of a single patient.

Decision for postoperative extubation, whether immediate or delayed, was taken based on anaesthetic considerations. All patients were shifted postoperatively to the paediatric ICU (PICU) for postoperative observation, irrespective of extubation status. All patients were treated with appropriate empiric antibiotics and analgesics initially, which were subsequently altered as per the pus culture and sensitivity results. Postoperative complications on immediate and long-term follow-up, such as non-resolution of fever, air leak, postoperative pneumonia, prolonged ICD requirement, prolonged requirement for ventilator support, residual disease, and recurrence of disease, were analysed.

Patients were followed up in the postoperative period with daily drain output monitoring and serial chest X-rays, apart from routine vitals monitoring and symptomatic improvement. ICDs were removed once output significantly decreased, air column movement stopped, and there was significant resolution of empyema on chest X-ray coupled with general clinical improvement, which was assessed by an experienced clinician/surgeon. 

Postoperatively, the patients were routinely followed up for a mean period of three months with monthly follow-up. During each monthly follow-up, a subjective assessment of the respiratory system by clinical examination of the chest was done. Routine, objective assessment of pleural status and lung expansion was done on the first follow-up using chest radiography only. In the absence of any symptoms and signs of any respiratory disease, a chest X-ray was only done on the first follow-up and omitted during the second and third follow-ups.

Statistical analysis

A data sheet was formulated to collect the data using Microsoft Excel version 2024 (Microsoft Corporation, Redmond, Washington, United States). All continuous data (e.g., age, duration of symptoms, amount of blood loss, duration of PICU stay, time for ICD removal) were calculated for mean±standard deviation and median (range). All categorical data (e.g., presence of postoperative fever, air leak, requirement for thoracotomy) were presented as frequency (percentages).

Ethical considerations

Ethical approval for the study was obtained from the Institutional Ethics Committee of Kalinga Institute of Medical Sciences (Ref. No: KIIT/KIMS/2262/2025). The case records, operative notes, and radiographic images were obtained from the medical records department with appropriate permissions and consent from patient guardians, the Radiology Department, and the Medical Superintendent. Informed consent was taken from parents after explaining the procedure and study inclusion. Patient confidentiality and anonymity were strictly maintained throughout data collection and analysis. All information was recorded in a structured proforma designed for the study.

## Results

**Figure 3:**
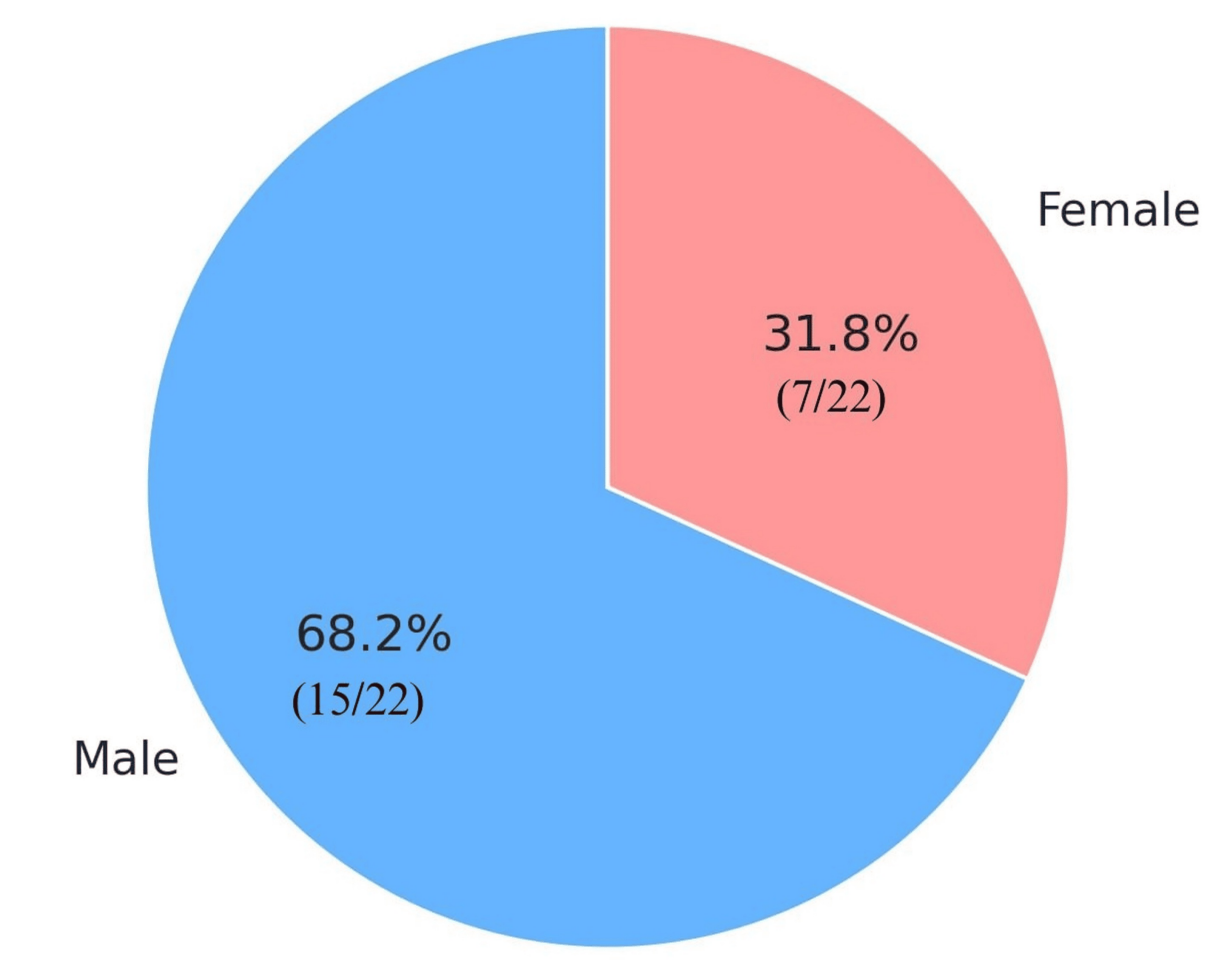
Distribution of the study population by sex

A total of 22 children were included in the study. The age distribution of the children ranged from two to 15 years, with a mean age at presentation of 7.4 years and a median of 7.5 years. Most patients were male, accounting for 15 out of the 22 cases (68.2%), while seven patients (31.8%) were female (Table 1, Figure 3).

**Table 1:** Demographic and preoperative characteristics of the patients (N=22) UTI: urinary tract infection; ICD: intercostal drain

Characteristics		Frequency (Percentage)
Sex	Male	15 (68.2%)
	Female	7 (31.85%)
Side of Empyema	Right-sided Empyema	15 (68.2%)
	Left-sided Empyema	7 (31.8%)
Concurrent Systemic Infections	Tuberculosis	1 (4.54%)
	Scrub Typhus	1 (4.54%)
	Typhoid Fever and UTI	1 (4.54%)
Congenital Anomalies	Unilateral Hydronephrosis	1 (4.54%)
	Congenital Inguinal Hernia	1 (4.54%)
Other Co-morbidities	Seizure Disorder	1 (4.54%)
Pre-operative Intervention Required	Preoperative ICD Insertion	13 (4.54%)
	Preoperative Intubation	1 (4.54%)

**Figure 4:**
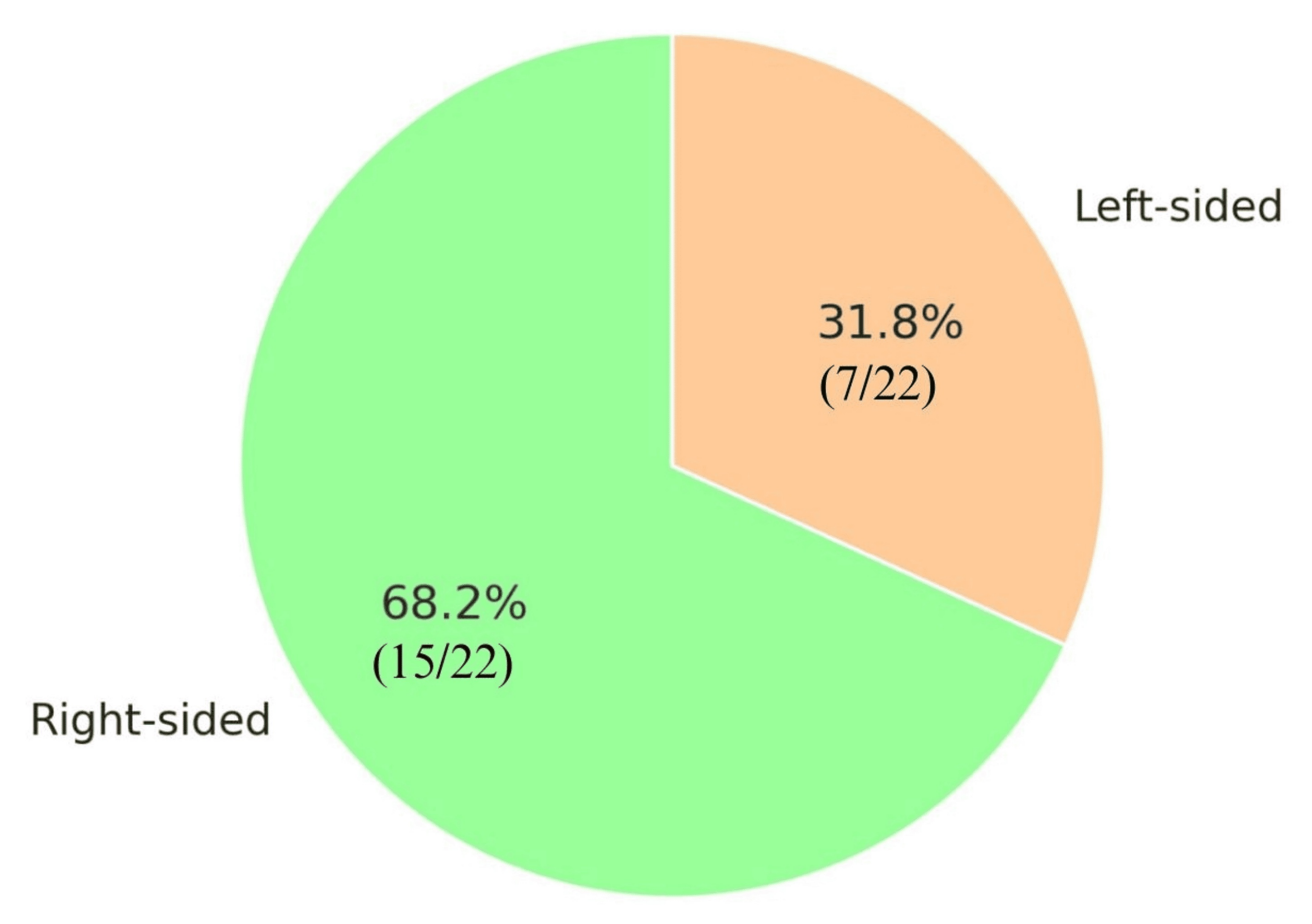
Distribution of the study population by the side involved

Right-sided empyema was observed in 15 patients (68.2%), while left-sided involvement occurred in seven patients (31.8%) (Figure 4). None of the patients in our study had bilateral empyema. However, two children had minimal contralateral side pleural effusion, which was managed with conservative management. Three children had concurrent systemic infections: one with tuberculosis, one with scrub typhus, and another with typhoid fever and a urinary tract infection. Two patients (9.1%) had congenital anomalies, including one with unilateral hydronephrosis and another with a congenital inguinal hernia. One child (4.5%) had a history of seizure disorder, as mentioned in Table 1.

**Figure 5:**
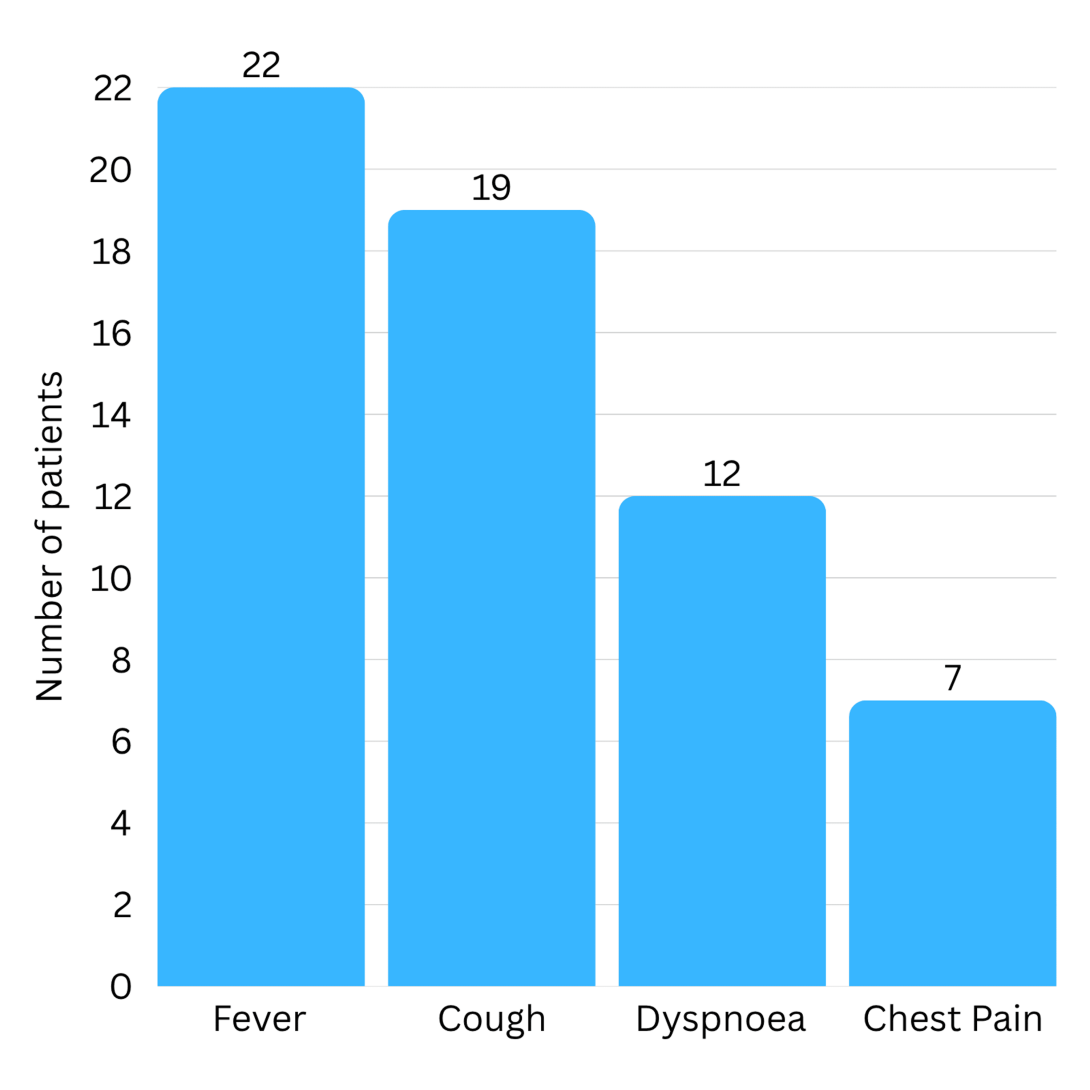
Frequency of presenting symptoms

Table 2 depicts that the most frequent presenting complaint was fever, reported in all 22 cases. Cough was noted in 19 children (86.4%), dyspnoea in 12 (54.5%), and chest pain in seven (31.8%) (Figure 5). On average, symptoms lasted 14.5 days before the surgery, with a range of 9-30 days. This includes the time from the onset of presentation to hospitalisation until the day of surgery. Preoperative ICD was inserted in 13 patients (59.1%), and only one child (4.5%) needed preoperative intubation.

**Table 2:** Presenting symptoms and duration in the study population (N=22)

Symptom and Duration	Value
Fever, n (%)	22 (100%)
Cough, n (%)	19 (86.4%)
Dyspnoea, n (%)	12 (54.5%)
Chest Pain, n (%)	7 (31.8%)
Duration of Symptoms before Surgery (days), mean ± SD	14.5 ± 5.2
Duration between Hospitalisation to Surgery (days), mean ± SD	3.1 ± 1.1
Duration of Symptoms before Surgery (days), range	9-30

**Figure 6:**
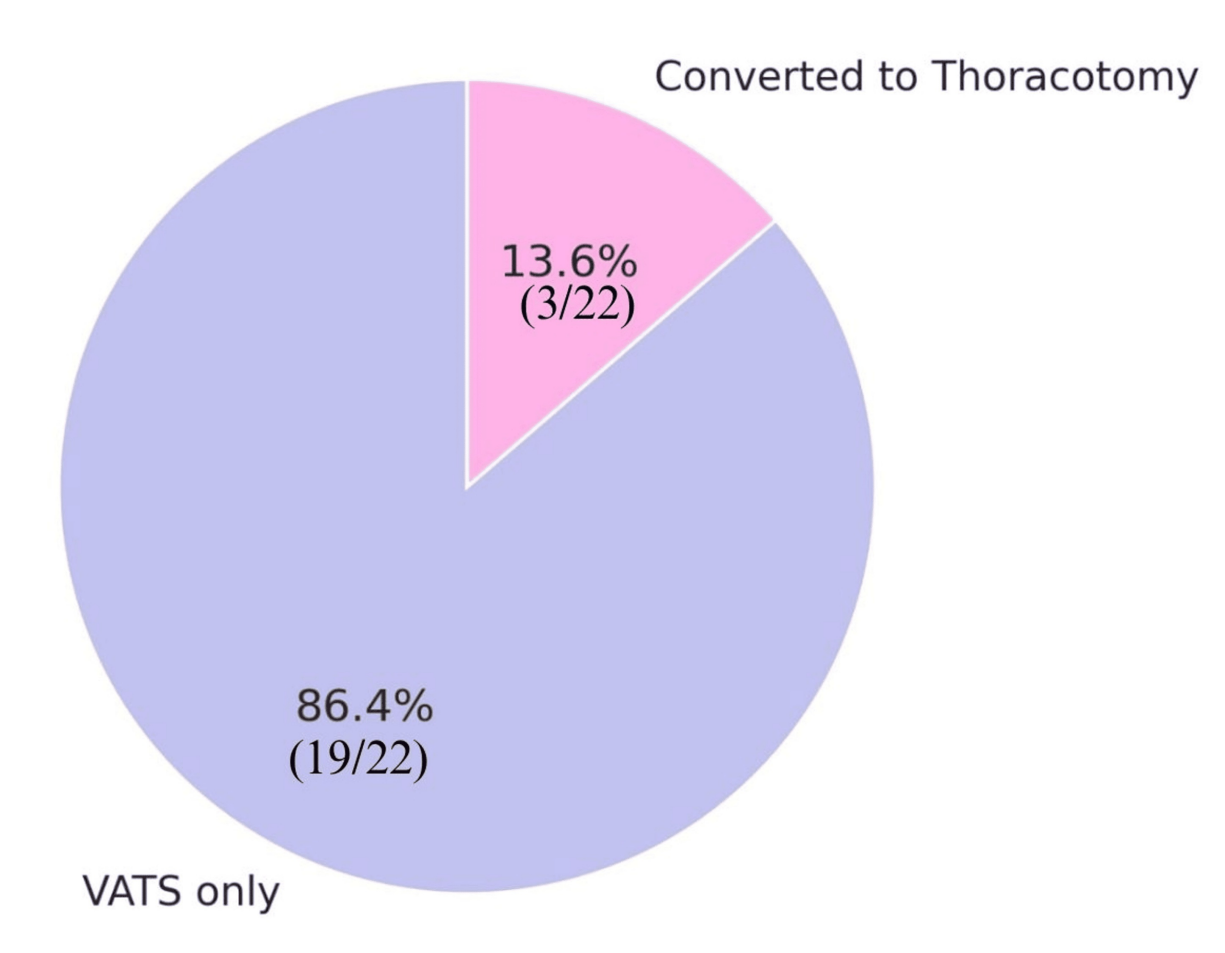
Distribution of the study population by operative procedure VATS: video-Assisted thoracoscopic surgery

**Figure 7:**
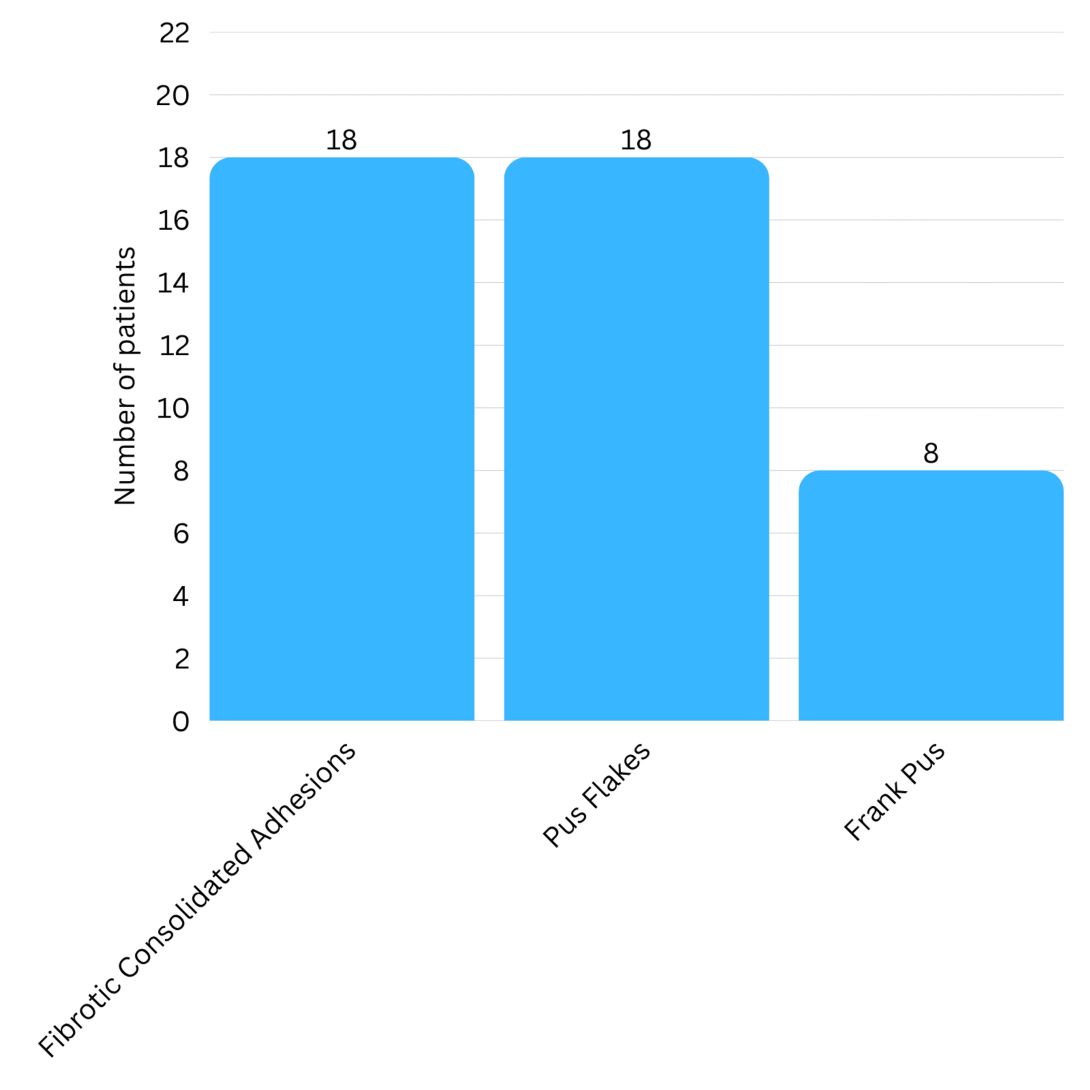
Intraoperative findings

Table 3 shows that VATS was the main surgical procedure for 19 patients (86.4%). In three cases (13.6%), the surgery involved conversion to an open thoracotomy due to dense pleural adhesions and pus flakes, which could not be removed via thoracoscopy (Figure 6). Pus flakes and fibrotic adhesions were noted in 18 patients (81.8%). Frank pus was noted in eight patients (36.4%) (Figure 7). The mean operative time was 96.1 minutes (range 40-160 minutes). Estimated blood loss during surgery averaged 60.2 mL (range, 20-100 mL). One patient had an iatrogenic injury during the procedure, a rent in the diaphragm, which was identified and repaired during the surgery without complications.

**Table 3:** Intraoperative findings and surgical details in the study population (N=22) Data presented as n (%) unless otherwise indicated.

Characteristic	Value
Conversion to Open Thoracotomy	3 (13.6%)
Intraoperative findings	
Pus flakes	18 (81.8%)
Fibrotic/Consolidated Adhesions	18 (81.8%)
Frank Pus	8 (36.4%)
Operative Time (minutes), mean ± SD	96.1 ± 32.8
Operative Time (minutes), median (range)	97.5 (40-160)
Estimated Blood Loss (mL), mean ± SD	60.2 ± 24.2
Estimated Blood Loss (mL), median (range)	65 (20-100)

**Figure 8:**
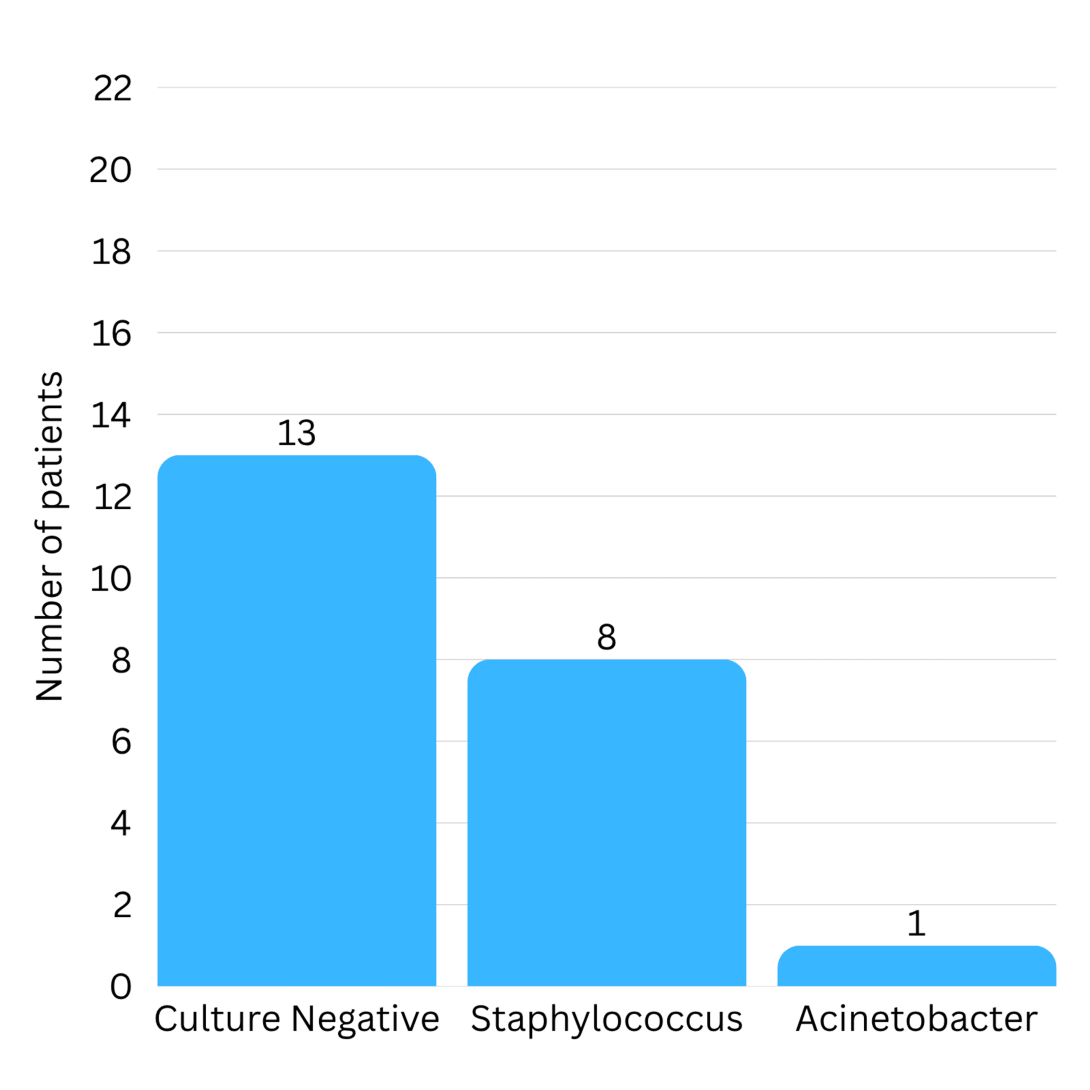
Microbiological findings

Results of microbiological and histopathological examination are summarised in Table 4. Microbiological analysis of the samples collected intraoperatively showed that pus cultures were negative in 13 cases (59.1%). *Staphylococcus* species were identified in eight patients (36.4%), and *Acinetobacter* was found in one case (Figure 8). Gram stain revealed pus cells in 17 samples (77.3%). Acid-fast bacilli were detected on Ziehl-Neelson staining in one case, and two cases tested positive for tuberculosis on the cartridge-based nucleic acid amplification test (CBNAAT). Histopathological examination showed inflammatory and granulation tissue in 20 patients (90.9%), while two cases (9.1%) had caseating granulomatous inflammation, consistent with tuberculosis.

**Table 4:** Microbiological and histopathological findings CBNAAT: cartridge-based nucleic acid amplification test

Investigation	Finding	Number of Samples
Pus Culture	Negative	13 (59.1%)
	*Staphylococcus* species	8 (36.4%)
	Acinetobacter	1 (4.54%)
Gram Stain	Pus cells	17 (77.3%)
	Organism (i.e. gram-negative coccobacilli)	1 (4.54%)
Acid-fast bacilli on Ziehl-Neelson staining	Positive	1 (4.54%)
	Negative	21 (95.46%)
CBNAAT for Tuberculosis	Positive	2 (9.1%)
	Negative	20 (90.9%)
Histopathology	Inflammatory and Granulation Tissue	20 (90.9%)
	Caseating Granulomatous Inflammation	2 (9.1%)

Table 5 describes the postoperative outcomes. Eleven (50%) patients were extubated right after surgery. All patients, irrespective of extubation status, were shifted to the PICU postoperatively for close monitoring. For the children who were shifted to PICU in an extubated state, the mean time to extubation was 23.8 hours (range, 2-84 hours). One child required prolonged intubation and ultimately was tracheostomised. The child remained on a ventilator support until day 36 after surgery. The median duration of ICU stay was 5.5 days, averaging 8.5 days. The overall range of ICU stay was 2-28 days. Of our 22 cases, 20 had an ICU stay of less than seven days. However, two patients needed extended ICU stay, one due to a tracheostomy and prolonged ventilator support, and another for non-surgical medical illness (refractory seizure disorder).

**Table 5:** Postoperative outcomes of the study population (N=22) Data presented as n (%) unless otherwise indicated PICU: paediatric intensive care unit; ICD: intercostal drain

Outcome	Value
Immediate extubation	11 (50%)
Shifted to PICU in intubated state	11 (50%)
Time to extubation (hours), mean ± SD	23.8 ± 28.9
Time to extubation (hours), range	2-84
Required tracheostomy	1 (4.54%)
Duration of ICU stay (days), mean ± SD	8.5 ± 2.3
Duration of ICU stay (days), median (range)	5.5 (2-28)
ICU stay < 7 days	20 (90.9%)
Postoperative fever	10 (45.5%)
Air leak	2 (9.1%)
ICD removal time for 1st ICD (days), mean ± SD	3.1 ± 1.9
ICD removal time for 2nd ICD (days), mean ± SD	9.8 ± 2.3
ICD removal time (days), range	7-20
Discharged with ICD in place	1 (4.54%)
Satisfactory lung re-expansion on chest X-ray post-ICD removal	21 (95.5%)
Incomplete lung re-expansion on chest X-ray post-ICD removal	1 (4.54%)
Hospital stay postoperatively (days), median (range)	14.5 (10-41)
Hospital stay > 10 days (non-surgical causes)	4
Hospital stay > 10 days (surgical cause i.e. Persistent air leak)	1
Normal respiratory exam (1 month follow-up)	20 (90.9%)
Complete radiological resolution (1 month follow-up)	15 (68.2%)
Complete radiological resolution (3 month follow-up)	22 (100%)
No residual empyema noted	22 (100%)
No recurrent empyema noted	22 (100%)
No restrictive lung disease Noted	22 (100%)

Postoperative fever occurred in 10 children (45.5%), and air leaks were noted in two cases (9.1%). The ICD was removed on average 9.8 days after surgery (range, 7-20 days). One patient went home with the drain still in place because of a continuing air leak. The drain for this child was removed during the first follow-up visit.

After the drain removal, chest X-rays showed satisfactory lung re-expansion in 21 patients (95.5%). Only one patient, who had a tracheostomy and required prolonged ventilator support, showed incomplete re-expansion.

The median postoperative hospital stay was 14.5 days (range, 10-41 days). Five children had long hospital stays. In four cases, the stay extended beyond 10 days due to non-surgical causes. Of these, two children had a long stay due to underlying tuberculosis. The third child had non-refractory seizure disorder, which presented post-operatively and mandated close observation in the PICU and ward. This child was hence only discharged on the 35th postoperative day. In the fourth case, while the acute empyema resolved, the child subsequently developed non-specific acute respiratory distress syndrome (ARDS) and ventilator-associated pneumonia, which required prolonged ventilation. This child ultimately was tracheostomised and discharged only on the 41st postoperative day. Thus, medical causes contributed largely to our patients requiring prolonged hospital stays. Only one child had a longer stay related to surgical factors, i.e., persistent air leak, which was managed conservatively and observed. Eventually, this child was discharged with the ICD in situ. With the exception of these five cases, all cases were discharged within 10 days postoperatively.

Follow-up results showed ongoing and complete clinical and radiological recovery in all children. After one month, 20 (90.9%) patients had normal respiratory examinations, and 15 (68.2%) showed complete radiological resolution. Some patients had residual radiographic changes like haziness or patchy opacities, but these completely resolved by the third month. By the third follow-up, all 22 patients had normal respiratory examinations and full radiological clearance. No residual or recurrent empyema was noted during follow-up.

## Discussion

Empyema thoracis remains a significant complication of bacterial pneumonia in children, especially when the disease worsens despite initial treatment. Timely surgical intervention is often necessary in these cases to prevent further decline and long-term lung issues. In our experience, VATS has proven to be a reliable and effective choice for managing acute, non-resolving empyema in paediatric patients.

In this study, patients showed typical symptoms, including fever (100%) and cough (86.4%), over half reported shortness of breath (54.5%), and a few children reported chest pain (31.8%). This is in line with the common presentations of empyema thoracis documented in other studies [5-7]. The similar patterns of presentation over the last decade reflect that not much has changed with respect to disease presentation and patterns of this disease.

As per Singh et al, most patients usually undergo VATS within two weeks of the onset of symptoms [3]. In our study, the mean duration of symptoms from presentation to surgery was 14.5 days, of which the mean duration of symptoms at the time of admission was 10.5 days. The long duration of symptoms prior to admission in our centre can possibly be attributed to the fact that it is an apex tertiary health care centre. Thus, the majority of the patients we receive were inadequately treated or referred following failure of medical management of their respiratory illness from peripheral hospitals. Due to the later presentation of these children, they presented to us in Stage II / III of acute empyema. However, prompt decision making on behalf of the treating team is reflected in the mean time between admission and surgery, which was 3.1 days.

Intraoperative findings in acute empyema, such as dense adhesions, pus flakes, and frank pus, can modify the surgical management strategy from VATS to an open thoracotomy. Conversion from VATS to open thoracotomy was done in three out of 22 of the cases (13.6%) owing to dense adhesions that could not be removed thoracoscopically. Open thoracotomy is associated with increased morbidity due to increased blood loss, larger incision, more postoperative pain, and increased risk of infection. However, the delayed presentation of the patients to our setup made decortication of severely thickened pleura nearly impossible without risking injury to bronchioles, lung parenchyma, and lung tissue. Hence, in such cases that cannot be managed even by VATS, prompt intraoperative decision-making for conversion to an open procedure is key. This is often the safer and more effective method. 

The median duration of postoperative hospital stay is comparable with other studies, which ranged from 4.9 days to 14.5 days [5,6,8]. In our study, the median duration of postoperative hospital stay was 14.5 days. Total postoperative length of stay was less than 10 days for 77.2% of our participants. However, of the remaining cases, non-surgical causes due to concurrent infections and prior comorbidities, such as tuberculosis and seizure disorder, contributed as a major reason for prolonged hospital stay. The longer hospital stay for these patients also significantly skewed the total median postoperative hospital stay duration in our study. One patient, despite resolution of empyema, was unable to be weaned off ventilator support and required prolonged ventilation, possibly due to the development of ventilator-associated pneumonia or unrelated ARDS. In such cases, no further surgical indication for hospitalisation was noted. One patient developed a postoperative air leak requiring prolonged observation in the hospital. This complication was diagnosed in the drain tube but did not contribute to any significant respiratory distress or cause any symptomatic discomfort to the patient. Due to non-resolution of the air leak with observation, the child was subsequently discharged with the ICD-in situ, which was removed on the first follow-up visit when the air leak had subsided. 

Intrapleural fibrinolytic therapy has also been used to treat empyema when intercostal drainage tube insertion alone is insufficient in clearing the pleural cavity. This therapy aims to break up septations and improve drainage [9]. Fibrinolytic agents such as alteplase and streptokinase are inserted into the pleural cavity via the intercostal drain and allowed to act, and clinico-radiological improvement of the patient is observed. Of note at this point is that while fibrinolytic therapy has shown some promise in the resolution of empyema, the response to this therapy remains highly unpredictable [10]. Our study reaffirms that the duration of hospital stay in case of primary operative intervention in the form of VATS or thoracotomy is significantly lower than that noted following the use of intercostal chest tube drainage alone or following fibrinolytic therapy, as reported in many reviews and meta-analyses [11-14]. A systematic review by Gates et al. revealed that postoperative mean length of stay for patients with primary VATS was 10.5 days, while for those with fibrinolytic therapy alone, it was 18.9 days [11]. Results of another review by Pacilli et al. were in favour of VATS over fibrinolytic therapy with respect to post-operative length of stay [12].

Also, initial operative intervention is associated with quicker recovery, lower rates of treatment failure, and less need for re-intervention. For instance, the re-intervention rate in the study by Pacilli et al. following VATS was 9.6%, and following fibrinolytic therapy was 22.5% [8,12,15,16]. This can be attributed to the fact that, compared to traditional management using intercostal drainage and fibrinolytic agents, VATS and/or thoracotomy provide better visualization of the pathology and allow for thorough debridement and decortication. Fibrinolytic therapy, although non-invasive, carries the risk of residual or recurrent disease, which can increase the overall morbidity from the disease. Such cases of empyema that don’t resolve with fibrinolytic therapy ultimately end up requiring a ‘rescue’ VATS.

Our postoperative outcomes were largely positive. Lung expansion was satisfactory in nearly all patients, and complications like prolonged air leaks or postoperative fever were relatively rare and manageable. Importantly, we did not observe any cases of residual or recurrent empyema during follow-up. These results support earlier studies suggesting that early surgical intervention not only leads to quicker symptom resolution but also contributes to excellent long-term lung recovery [8,17].

The use of VATS in paediatric empyema has developed over time, with growing evidence supporting its role as the main method, especially in stages II and III of the disease [18,19]. Timing is crucial-delays in surgery have been linked to greater operative complexity, higher conversion rates, and longer recovery periods [20].

Limitations of this study include that it was a retrospective single-centre study with a small sample size. There is a need for multicenter prospective randomized controlled trials to further study the effectiveness of various treatment modalities for paediatric acute empyema. Overall, our experience adds to the increasing evidence that VATS is a safe, effective, and well-tolerated procedure for children with complicated empyema. It provides a minimally invasive option with clear benefits in both the short and long term. By recognizing clear clinical and radiological signs for surgery and acting early, we can improve outcomes and lessen the impact of these challenging conditions.

## Conclusions

VATS is a safe and effective method for managing stage II and III acute empyema in children. This is also true for cases of stage I empyema that do not respond to medical treatment and intercostal drainage. It is associated with faster recovery, shorter length of hospital stay, and almost nil rate of re-intervention compared with other less invasive methods of treatment of acute empyema. Further, development of safe practices with respect to the VATS procedure has minimised the rates of conversion of surgery to a more invasive open thoracotomy unless absolutely indicated. Early surgical intervention, based on clinical judgment and imaging results, seems vital for achieving the best recovery.

Our experience supports existing studies that promote VATS as a primary surgical choice for stages II and III paediatric empyema. While considering individual patient factors and the stage of the disease is important, performing VATS in a timely manner can significantly shorten hospital stays, improve recovery after surgery, and reduce long-term issues. Future studies with larger groups could help clarify when surgery is needed, leading to even better outcomes for children facing this condition.
